# Impact of switching from the originator adalimumab to a biosimilar: a retrospective cohort study

**DOI:** 10.1186/s12865-025-00693-9

**Published:** 2025-07-03

**Authors:** W. H. A. van Poecke, N. E. F. Hooi, T. K. Mossel, M. A. W. Hermans, P. L. A. van Daele, E. M. Bunnik, Z. Brkic, L. K. Sels, A. A. H. J. Thiadens, P. M. van Hagen, J. A. M. van Laar, S. M. Rombach

**Affiliations:** 1 Department of Internal Medicine, Allergy and Clinical Immunology, Erasmus MC University Medical Center, Rotterdam, the Netherlands; 2 Department of Pharmacy, Erasmus MC University Medical Center, Rotterdam, the Netherlands; 3 Department of Medical Ethics, Philosophy, and History of Medicine, Erasmus MC, Rotterdam, Netherlands; 4 Department of Ophthalmology, Erasmus MC University Medical Center, Rotterdam, the Netherlands

**Keywords:** Flare, adverse effects, biosimilar, adalimumab, Hyrimoz, Humira

## Abstract

**Introduction:**

Adalimumab is a monoclonal antibody that is used to treat autoimmune and inflammatory diseases. Biosimilars for adalimumab, including Hyrimoz, have been developed. We aimed to evaluate the effectiveness and adverse effects of Hyrimoz after switching.

**Methods:**

The cohort consisted of patients treated with adalimumab at the Clinical Immunology Outpatient Department of the Erasmus Medical Center between February 2021 and February 2023. Data were collected through electronic patient files and questionnaires sent to the patients. The primary outcome was the number of flares after switching to Hyrimoz, compared to a similar period before the switch. The secondary outcomes were reported adverse effects and patient experience using Hyrimoz.

**Results:**

A total of 185 patients were eligible for inclusion. There was no significant difference in the occurrence of flares between Humira and Hyrimoz (*P* = 0.456). Forty-six of the 185 patients reported adverse effects (24.9%). A total of 25/185 (13.5%) patients reported pain during injection, which was the most frequently reported adverse effect. During the course of this study, 60/185 (32.4%) patients discontinued Hyrimoz treatment because of flares (*n* = 17 [9.2%]), adverse effects (*n* = 27 [14.6%]), or more subjective complaints (*n* = 15 [8.1%]) related to the underlying disease. One patient discontinued treatment because of inactive disease.

**Conclusion:**

The number of flares before and after switching to Hyrimoz was comparable. However, adverse effects and increased subjective complaints have been reported after switching to this new biosimilar.

**Supplementary Information:**

The online version contains supplementary material available at 10.1186/s12865-025-00693-9.

## Introduction

Biological drugs or biologics, such as monoclonal antibodies, are advantageous for the treatment of immune-mediated diseases (IMIDs) and have been used worldwide since their invention in 1975 [1, 2]. As conventional disease-modifying anti-rheumatic drugs (DMARDs) are still used as initial or maintenance therapies for many autoimmune diseases, monoclonal antibodies are commonly used as second- or third-line therapies. Monoclonal antibodies are highly effective and generally well-tolerated because of their selective targeting [3]; however, they are expensive, which has led to the development of biosimilars [4], which are attempted copies of a biological drug, but are often slightly different in pharmacodynamic and pharmacokinetic properties because of changes in glycosylation [4]. This is due to the unique and complex structure of glycoproteins and the use of different cell systems to produce monoclonal antibodies.

Although biosimilars are expected to have the same efficacy, their effectiveness may differ slightly in clinical practice [4]. New biosimilars have been developed for adalimumab (Humira) [5]. The monoclonal antibody ‘adalimumab’ binds to TNF-alpha, neutralizing the pro-inflammatory effects of TNF-alpha [6–9]. The first studies on IMIDs showed the positive effects of adalimumab, especially in patients with rheumatoid arthritis [10–12]. Since then, it has proven to be highly effective in the treatment of many other immune mediated diseases, including psoriatic arthritis, ankylosing spondylitis, Crohn’s disease, plaque psoriasis, uveitis, sarcoidosis, and Behçet’s disease [11]. Although new monoclonal antibodies have been developed for these diseases, adalimumab remains one of the main treatment options for various IMIDs [11, 13].

Humira® is one of the best selling drugs worldwide, but is also costly [14]. Research in 2013 in the United Kingdom estimated the cost of Humira in patients with Crohn’s disease be around GBP £15,062 per patient per year [15]. Nowadays the price of Humira is much lower but it is still expensive. As Humira’s patent expired, biosimilars were developed [16–18], such as GP2017, which has been tested for safety and efficacy [17].

No differences in clinical outcomes between Humira® and GP2017 in patients with rheumatoid arthritis were shown, making GP2017 a safe and effective replacement for Humira® when treating rheumatoid arthritis. In addition, a study by Blauvelt et al. compared Humira and GP2017 in patients with psoriasis, and showed similar efficacy and safety of GP2017 in these patients [16].

One of the first studies evaluating the effectiveness of GP2017 in inflammatory bowel disease showed that GP2017 is a safe and effective treatment for this indication [19].

The same study group showed that other biosimilars of adalimumab are effective and well tolerated in patients with Crohn’s disease and switching between different biosimilars of adalimumab did not change treatment outcome [20, 21]. A major advantage of a biosimilar is its low price. The price of GP2017 (Hyrimoz) is significantly lower than that of its predecessor [22].

Hyrimoz was, therefore, introduced as a treatment option at the Erasmus Medical Center, Rotterdam. The department of immunology at the Erasmus Medical Center is a tertiairy referral center for various immune mediated diseases, with over more than 4000 patients. Some diagnoses are particularly rare. Anti-TNF is prescribed for various registered indications and sometimes off label indications. In this observational study, we aimed to monitor and evaluate the effectiveness and possible adverse effects of switching from the originator adalimumab to a biosimilar of adalimumab (Hyrimoz) in clinical practice.

## Methods

### Data collection

At the beginning of 2022, patients at the Erasmus Medical Center treated with adalimumab (Humira), were switched to Hyrimoz by the hospital pharmacy, based on similar reported safety and efficacy. Patients treated with Hyrimoz at the Clinical Immunology Department were included in this observational clinical cohort study, identified using the medication registry of the pharmacy at the Erasmus Medical Center.

All patients who used Hyrimoz and had checkups at the Clinical Immunology Department in the year before switching to Hyrimoz were eligible for inclusion. Patients were monitored for the year after switching. The follow-up period started 1 year before the switch and ended 1 year thereafter. Data were collected by evaluating electronic patient files and questionnaires that were sent to all patients (available on request). Approval was obtained from the Medical Ethics Committee (MEC-2022–0500).

### Patients and methods

Patients and health care professionals were informed by the colleagues of the pharmacy that the medication was switched because the market protection of adalimumab ( Humira) was expired and a biosimilar was available with a supposed similar effectiveness but with lower costs.

**Fig. 1:**
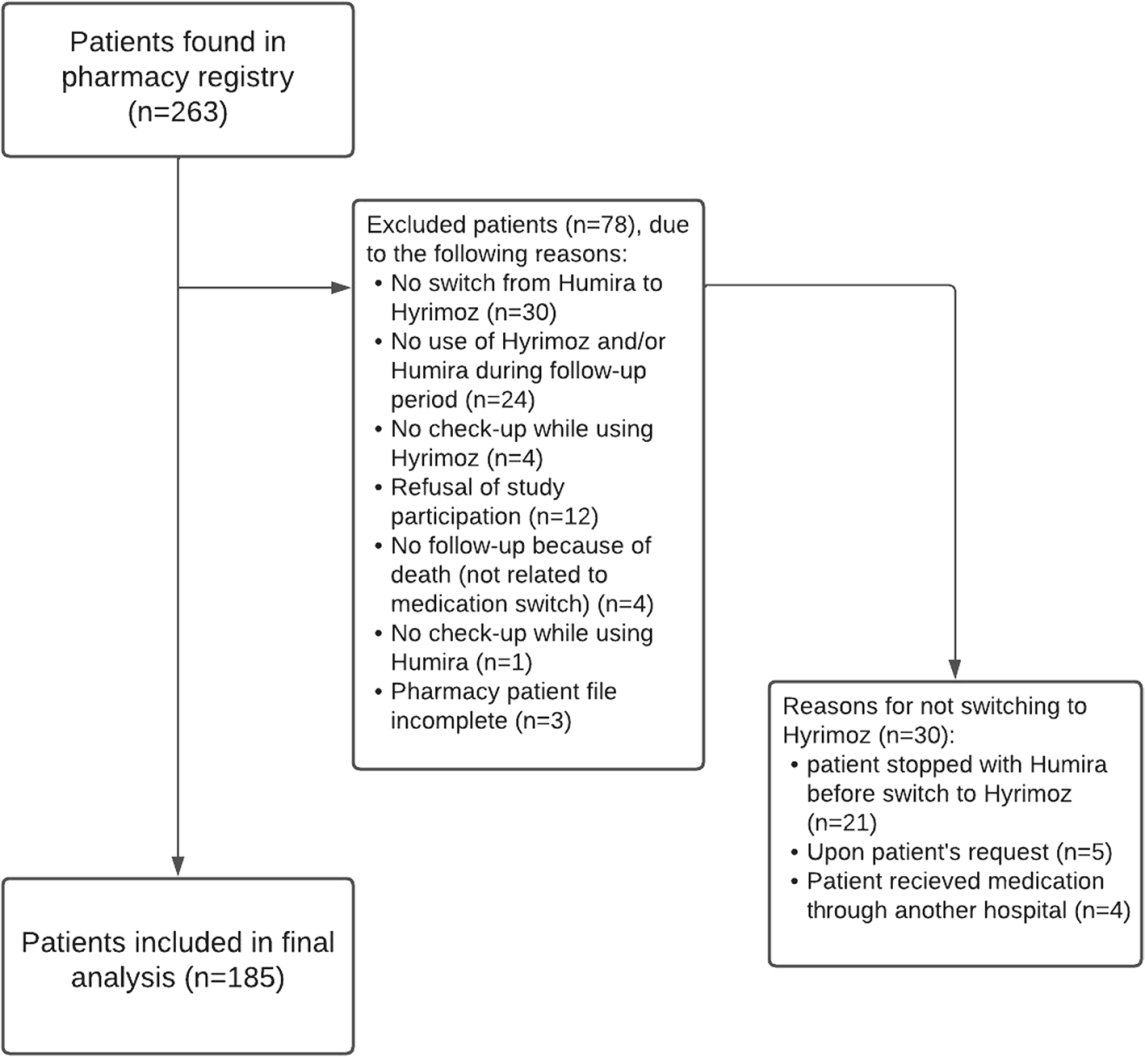
Flow chart of the inclusion process

As the pharmacy changed their purchasing policy, all patients received the biosimilar, unless the patient or medical doctor specifically requested the biological which was because of various reasons (Fig. 1). For further support and instructions on how to use the new medication, patients were referred to their medical doctor or nurse from the immunology department.

The patients included in this study were treated with adalimumab at the Department of Clinical Immunology for various IMIDs. At regular outpatient visits, the patients’ health status was checked and the effects of therapy were monitored, including whether there were symptoms indicating a flare, medication compliance, and any adverse effects. At the start of treatment, the patients were trained to inject adalimumab subcutaneously for continued use at home. When the patients’ stock of Humira ran out, they were switched to Hyrimoz under the guidance of the pharmacy.

Both Humira as well as Hyrimoz were available as pre-filled syringes as well as autoinjectors and were prescribed based on the patient’s preference. In case a patient preferred another form of injection, this could be changed.

Humira injections did not contain citrate. Hyrimoz injections contained citrate as an additive.

The switch was implemented by the pharmacy in January 2022.

In the case of a flare or when the patient experienced unacceptable adverse effects, a personalized treatment plan was created as follows: 1) continuing Hyrimoz with or without additional medication; 2) switching from Hyrimoz to Humira; or 3) starting another immune-mediating drug.

### Outcome measurements

The primary outcome of this study was the number of flares that occurred in the years before and after the switch from Humira to Hyrimoz. In cases where a patient used Humira for less than 1 year, the follow-up time from the start of Humira was used (Table 1). A flare was defined as objective inflammation assessed by a treating physician from the Clinical Immunology Department. Depending on the clinical symptoms of the flare, they were objectified using laboratory investigations and/or X-ray/CT scans, if possible. In case of any doubts of a flare the treating physician was contacted and if there were no objective signs of a flare, this was counted as a subjective symptom instead of a flare.

**Table 1:** Patient characteristics, total cohort

Number of patients, N (%)	185 (100%)
Sex, N (%)	
Male	82 (44.3%)
Female	103 (55.7%)
Median age, years (range)	52 (21–86)
Reason for adalimumab use, N (%)	
Uveitis associated with systemic disease	60 (32.4%)
Isolated uveitis	41 (22.2%)
M. Behçet	37 (20.0%)
(Neuro)sarcoidosis	23 (12.4%)
Relapsing polychondritis	2 (1.1%)
Morbus Sjögren	1 (0.5%)
Lupus erythematodes	1 (0.5%)
X-linked agammaglobulinemia	1 (0.5%)
Aphthous stomatitis	2 (1.1%)
Hidradenitis suppurativa	1 (0.5%)
Systemic sclerosis	1 (0.5%)
Lupus mastitis	1 (0.5%)
PAMI syndrome	1 (0.5%)
Presumed Ocular Histoplasmosis Syndrome	2 (1.1%)
Scleritis	3 (1.6%)
Crohn’s disease	1 (0.5%)
Erdheim Chester	1 (0.5%)
Shulman’s syndrome	1 (0.5%)
H-syndrome	1 (0.5%)
Dermatomyositis	1 (0.5%)
TBK-1 deficiency	1 (0.5%)
Susac syndrome	2 (1.1%)
Undefined hyperinflammation	1 (0.5%)
Cerebral vasculitis	1 (0.5%)
Auto-inflammatory syndrome	1 (0.5%)
Stevens–Johnsen syndrome	1 (0.5%)
Median number of checkups (range)	5 (2–11)
Median treatment duration for the use of Humira, weeks (range)	45 (1–78)
Median treatment duration for the use of Hyrimoz, weeks (range)	33 (1–60)
Median treatment duration for the use of Humira after switching back from Hyrimoz	15 (4–38)
Use of co-medication	
Azathioprine	16 (8.6%)
Colchicine	16 (8.6%)
Dexamethasone	1 (0.5%)
Mycophenolate mofetil	4 (2.2%)
Prednisone	33 (17.8%)
MTX	45 (24.3%)
Plaquenil/hydroxychloroquine	11 (5.9%)
Apremilast	2 (1.1%)
Dapsone	2 (1.1%)
Mycophenolic acid	2 (1.1%)
Quinsair	1 (0.5%)

In cases of uveitis, a flare was determined by an ophthalmologist and classified according to the SUN criteria [23]. A flare was defined as active uveitis in one or both eyes, if there was inactive disease at the previous check-up. The secondary outcomes of this study were the reported adverse effects and subjective symptoms. Subjective symptoms were classified as aggravated symptoms related to the underlying disease, which could not be objectived as a flare by the treating physician.

This information was obtained from questionnaires and/or electronic patient files.

### Statistical analysis

Patient characteristics were analyzed and presented as descriptive data. As the included patients were their own controls, no significant differences in the characteristics between the two groups were expected. Differences in flares between the use of Humira and Hyrimoz and patient characteristics of patients with flares were compared using a Wilcoxon signed-rank test. The follow-up times for both Humira and Hyrimoz were calculated to evaluate the absence of major discrepancies during the study period. Continuous variables were compared using the Mann–Whitney U. Dichotomous data were compared using Fisher’s exact test. All statistical analyses were performed using IBM SPSS Statistics for Windows, Version 28.0.1.0 Armonk, NY. Statistical significance was set at *P < *0.05.

## Results

### Patient characteristics

The data of 263 patients were retrieved from the database and acquired from the outpatient pharmacy. Of the 263 patients, 78 were excluded, as shown in Fig. 1. A total of 185 patients who switched from Humira to Hyrimoz were included in the study (Table 1). All patients underwent checkups at the Department of Immunology. Electronic patient files from 1 January 2021, 1 year before switching, to 17 February 2023, approximately 1 year after switching, were reviewed. The median treatment duration for Humira was 45 weeks (range 1–78 weeks) and that for Hyrimoz was 33 weeks (range 1–60 weeks) (*p < *0.001). The median follow-up period for patients who switched back to Humira after using Hyrimoz was 15 weeks (range 4–38 weeks), shorter than the follow-up period in the Hyrimoz study (*p* = 0.008). The most prevalent indications for the use of adalimumab were systemic disease with uveitis (32.4%), isolated uveitis (22.2%), Behçet’s disease (without active uveitis for which treatment was indicated) (20.0%) and (neuro)sarcoidosis (12.4%) (Table 1). The median age of the patients was 52 (range 21–86) years, and 55.7% were female. The median number of checkups in the hospital was five (range 2–13) over the 2 years of follow-up.

### Patient characteristics from questionnaires

All 185 patients received an email containing a questionnaire. Of the 185 patients, 105 (56.8%) completed the questionnaire (Table 2).

**Table 2:** Patient characteristics of patients that filled in the questionnaire

Number of patients, N (%)	105 (100%)
Sex, N (%)	
Male	46 (43.81%)
Female	59 (56.19%)
Median age, years (range)	54 (23–86)
Reasons for adalimumab use, N (%)	
Uveitis based on systemic disease	37 (35.2%)
Isolated uveitis	26 (24.8%)
(Neuro)sarcoidosis	16 (15.2%)
M. Behçet without uveitis	13 (12.4%)
Aphthous stomatitis	2 (1.9%)
Scleritis	2 (1.9%)
Susac syndrome	1 (0.95%)
Cerebral vasculitis	1 (0.95%)
Auto-inflammatory syndrome	1 (0.95%)
Stevens–Johnsen syndrome	1 (0.95%)
Lupus erythematodes	1 (0.95%)
X-linked agammaglobulinemia	1 (0.95%)
Systemic sclerosis	1 (0.95%)
Lupus mastitis	1 (0.95%)
Presumed Ocular Histoplasmosis Syndrome	1 (0.95%)
Origin, N (%)	
Asian	3 (2.86%)
European	67 (63.81%)
North-Africa	5 (4.35%)
South American	2 (1.90%)
Turkish	3 (2.86%)
Not reported	25 (23.81%)

### Difference in flares

#### Total cohort

**Fig. 2:**
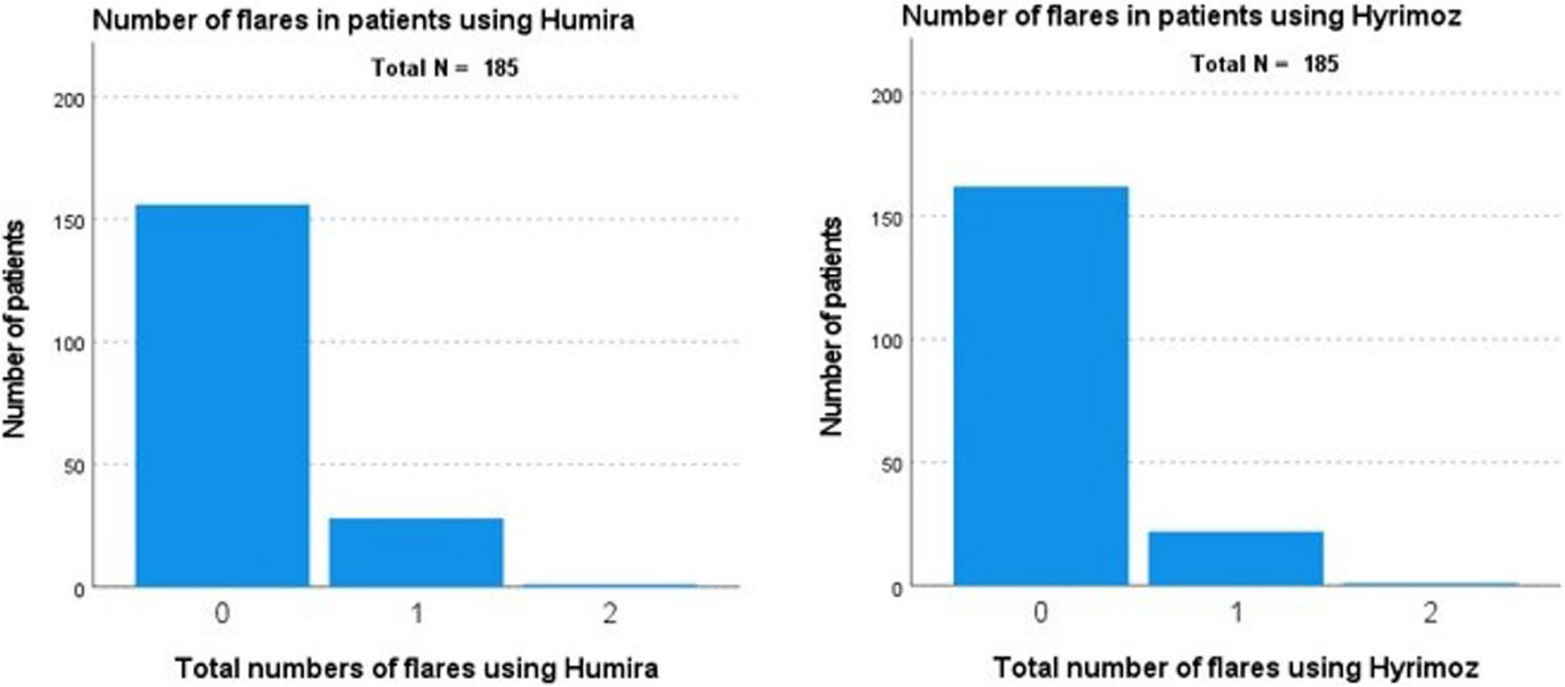
Occurrence of flare during use of Humira (left) and Hyrimoz (right)

All 185 patients had accessible records containing information on the occurrence of flares during the follow-up period. Table 3 shows the characteristics of individuals with one or multiple flares during the follow-up period. During the follow-up period for Humira, 29 flares were reported in 28 patients in the year prior to switching to Hyrimoz. Twenty-four flares were reported in 23 patients after the switch; Fig. 2 shows the occurrence of these flares at Humira and Hyrimoz. There was no difference in the number of flares before and after switching from Humira to Hyrimoz (*p* = 0.456) throughout the follow-up period of this study.

**Table 3:** Clinical characteristics of patients that suffered from flares

	Flare during Humira use*	Flare during Hyrimoz use*
Number of patients	28	23
Number of flares during follow-up	29	24
Females/males	17/11	12/11
Median age in years (range)	46.5 (21–78)	45 (27–86)
Follow-up time in weeks (range)	51 (17–78)	34 (6–56)
Diseases in which the flare occurred:		
-Uveitis without systemic disease	3	0
-Uveitis (flare) in case of systemic disease	0	5
-M. Behçet	15	12
-(Neuro)sarcoidosis	4	4
-Auto-inflammatory syndrome	1	-
-Relapsing polychondritis	1	1
-Lupus erythemadoses	1	-
-PAMI syndrome	1	-
-Scleritis	1	1
-Colitis ulcerosa	1	-
In the past 3 months before flare occurred:		
Reduction of:		
-Topical steroids	-	-
-Steroids intravitreal and/or systemic	-	2
-DMARD (methotrexate, azathioprine, mycofenolate mofetil)	-	1
-Other biological	-	1
Antibodies measurements in case of a flare	6 in 6 patients	14 in 14 patients
Antibodies present in case of a flare	0	1
Adalimumab level measurements	6 in 6 patients	14 in 14 patients
Adalimumab level decreased (compared to reference level)	in 2 patients	in 7 patients
Number of flares in the previous year before switch	Not applicable	6 in 6 patients

^*^There was no difference in the number of flares before and after switching from Humira to Hyrimoz (*p* = 0.456) throughout the follow-up period of this study

#### Subgroup: patients with uveitis

In total, 101 patients used adalimumab because of uveitis (in the past). Performing a subgroup analysis in this patient group, 3 of the 101 patients with uveitis developed a flare before switching and 5 of the 101 patients developed a flare after switching (*p* = 0.366). Of these patients, none suffered a flare both before and after switching to Hyrimoz.

#### Patients with and without a flare during Hyrimoz treatment

Patients who experienced a flare during Hyrimoz treatment and those who were free of flares were compared to evaluate the potential differences in characteristics. No significant differences in sex, age, or reason for adalimumab use were identified between the two groups. In the group that experienced a flare during Hyrimoz treatment, there were more reported adverse effects than in the group that did not experience a flare (*P* = 0.009) (Table 4). The most reported adverse effect, pain of the injection, was reported more frequently in the group that experienced a flare during Hyrimoz use than in the group without flares (*p* = 0.001).

**Table 4:** Clinical characteristics of patients that suffered a flare during use of Hyrimoz and patients that did not suffer a flare during use of Hyrimoz*

	No flare during Hyrimoz	Flare during Hyrimoz	
Number of patients, N (%)	162	23	-
Sex, N (%)			
Male	-71 (43.8%)	-11 (47.8%)	*P* = 0.823
Female	-91 (56.2%)	-12 (52.2%)	*P* = 0.823
Median age, years (range)	53 (21–84)	44.5 (27–86)	*P* = 0.032
Reason for adalimumab use, N (%)			
Uveitis based on systemic disease	51 (31.7%)	9 (37.5%)	*P* = 0.481
Isolated uveitis	41 (24.8%)	0 (0%)	*P* = 0.0039
M. Behçet without uveitis	28 (17.6%)	9 (34.6%)	*P* = 0.023
(Neuro)sarcoidosis	20 (12.6%)	3 (11.5%)	*P* = 1.000
Scleritis	2 (1.3%)	1 (3.8%)	*P* = 0.330
Relapsing polychondritis	1 (0.6%)	1 (3.8%)	*P* = 0.234
Other disease	19 (11.9%)	0 (0%)	-
Adverse effects, N (%)	47 (29.2%)	11 (45.8%)	*P* = 0.009
Painful injection	16 (15.5%)	9 (33.3%)	*P* = 0.001
Headaches or migraine	8 (5.0%)	1 (3.8%)	*P* = 1.000
Nausea	7 (4.4%)	0 (0%)	-
Cramps or muscle pain	5 (3.1%)	1 (3.8%)	*P* = 0.489
Illness and/or fever	3 (1.9%)	0 (0%)	-
Fatigue	7 (4.4%)	0 (0%)	-
Joint aches	5 (3.1%)	1 (3.8%)	*P* = 0.489
Skin complaints	3 (1.9%)	0 (0%)	-
Dizziness	4 (2.5%)	0 (0%)	-
Diarrhea	3 (1.9%)	0 (0%)	-
Numbness of the legs	1 (0.6%)	0 (0%)	-
Heart palpitations	1 (0.6%)	0 (0%)	-
Edema	1 (0.6%)	0 (0%)	-
Beep in ear	3 (1.9%)	0 (0%)	-
Blurriness of the eye	1 (0.6%)	0 (0%)	-
Complaints of extreme coldness	1 (0.6%)	0 (0%)	-
Lowered function of kidneys and lungs	1 (0.6%)	1 (3.8%)	*P* = 0.235
Epistaxis	1 (0.6%)	0 (0%)	-
Shortness of breath	1 (0.6%)	0 (0%)	-

^*^P-values were calculated in case adverse effects occurred in the flare group

#### Adverse effects of Hyrimoz

All 105 patients completed a questionnaire regarding possible adverse effects. Seventy-nine patients did not report any adverse effects; the remaining 26 patients experienced adverse events. Eighty patients did not complete the questionnaire; therefore, the electronic patient files of these patients were checked for possible adverse effects. In these records, 20 patients reported adverse effects to their physicians. In total, 46 of the 185 patients reported adverse effects (24.9%). The most commonly reported adverse effect was pain of the injection: 25/185 (13.5%). Eight patients experienced headaches or migraines after initiating Hyrimoz therapy. Nausea was reported in seven patients, fatigue in seven, cramps or muscle pain in five, and dizziness in four. The total variety of reported adverse effects are shown in Table 5.

**Table 5:** Reported side effects of Hyrimoz

Reported in questionnaire		Reported in electronic patient file		Total reported side effects	
Number of patients, N (%)	105 (100%)	Number of patients, N (%)	80 (100%)	Number of patients, N (%)	185 (100%)
No side effects, N (%)	79 (75.24%)	No side effects, N (%)	60 (75%)	No side effects, N (%)	139 (75.14%)
Side effects, N (%)	26 (24.76%)	Side effects, N (%)	20 (25%)	Side effects, N (%)	46 (24.86%)
Painful injection	11 (10.47%)	Painful injection	14 (17.5%)	Painful injection	25 (13.51%)
Headaches or migraine	6 (5.71%)	Headaches or migraine	2 (2.5%)	Headaches or migraine	8 (4.32%)
Nausea	5 (4.76%)	Nausea	2 (2.5%)	Nausea	7 (3.78%)
Cramps or muscle pain	3 (2.86%)	Cramps or muscle pain	2 (2.5%)	Cramps or muscle pain	5 (2.7%)
Illness and/or fever	3 (2.86%)	Epistaxis	1 (1.25%)	Illness and/or fever	3 (1.62%)
Fatigue	5 (4.76%)	Fatigue	2 (2.5%)	Fatigue	7 (3.78%)
Joint aches	3 (2.86%)	Joint aches	2 (2.5%)	Joint aches	5 (2.7%)
Skin complaints	1 (0.95%)	Skin complaints	2 (2.5%)	Skin complaints	3 (1.62%)
Dizziness	4 (3.81%)	Shortness of breath	1 (1.25%)	Dizziness	4 (2.16%)
Lowered function of kidneys and lungs	1 (0.95%)			Diarrhea	3 (1.62%)
Numbness of the legs	1 (0.95%)			Numbness of the legs	1 (0.54%)
Heart palpitations	1 (0.95%)			Heart palpitations	1 (0.54%)
Edema	1 (0.95%)			Edema	1 (0.54%)
Beep in ear	1 (0.95%)			Beep in ear	3 (1.62%)
Blurriness of the eye	2 (1.90%)			Blurriness of the eye	1 (0.54%)
Complaints of extreme coldness	1 (0.95%)			Complaints of extreme coldness	1 (0.54%)
Diarrhea	3 (2.86%)			Lowered function of kidneys and lungs	1 (0.54%)
				Epistaxis	1 (0.54%)
				Shortness of breath	1 (0.54%)

#### Discontinuation of Hyrimoz

Of the 185 patients included in this study, 60 (32.4%) discontinued Hyrimoz during the follow-up period. Of these 60 patients, one switched back to a different immunosuppressant, 17 stopped because of (the suspicion of) a flare, 15 experienced subjective symptoms of the underlying disease, and 27 experienced adverse effects. In 17/185 (9.2%) of these cases, the patients switched back to Humira because of (the suspicion of) a flare. Owing to the subjective symptoms of the disease, 15/185 (9.7%) patients stopped using Hyrimoz and switched back to Humira (*n* = 13), infliximab (*n* = 1), or tocilizumab (*n* = 1). Twenty-seven of the 185 patients (14.6%) stopped Hyrimoz treatment because of adverse effects, of which the painfulness of the injection was the most reported.

#### Follow-up after flare during Hyrimoz treatment

Twenty-three of the 185 patients (12.4%) experienced a flare during Hyrimoz treatment. Of these, 17 (73.9%) switched back to Humira after experiencing a flare, five continued Hyrimoz with or without immunosuppressant medication, and one patient stopped treatment with adalimumab and switched back to another immunosuppressant medication.

#### Follow-up in patients continuing Hyrimoz after a flare while using Hyrimoz

Of the five patients who experienced a flare and continued Hyrimoz, three patients each had serum adalimumab levels and antibodies measured, respectively. Adalimumab levels were decreased in all three patients; antibodies were detected in none. Two of these three patients reported not using Hyrimoz at an adequate frequency. One patient had no decreased levels of adalimumab, but also reported not using Hyrimoz at an adequate frequency. One patient received supplementary prednisone due to disease flares.

#### Follow-up in patients switching to Humira after a flare while using Hyrimoz

The characteristics of the 17 patients who returned to Humira use are presented in Table 6. In these patients developing a flare, serum drug levels were measured in 14 of the 17 patients using Hyrimoz. Serum levels were decreased in 6 of these 14 patients (42.9%). Anti-drug antibodies were detected in one of the 14 patients (7.1%). Of the 17 patients who switched back to Humira, three received additional immunosuppressant medication, two received prednisone, and one received kenacort.

**Table 6:** Follow-up after Hyrimoz flare with switchback to Humira

	**Flare during Hyrimoz**	**Follow-up after switching back to Humira because of a flare**
Number of patients	23	17
Number of flares during follow-up	24	1
Females/males	12/11	7/10
Median age in years (range)	45 (27–86)	49 (34–86)
Follow-up time in weeks (range)	34 (6–56)	15 (4–29)
Underlying disease:		
Uveitis in case of systemic disease	5	7
M. Behçet	12	6
Neuro(sarcoidosis)	4	3
Scleritis	1	1
Relapsing polychondritis	1	
Anti-drug antibodies measured	14	5
Anti-drugantibodies present	1	1
Adalimumab level measured	14	5
Adalimumab level below detection limit	7	3
Co-medication after Hyrimoz flare	NA	3
Type of co-medication:		
Prednisone		2
Kenacort		1

Topical steroids were used in cases of flare of uveitis, based on the discretion of the ophthalmologist. One patient developed a flare during follow-up while being re-treated with Humira (Table 6). However, the follow-up period was relatively short.

**Fig. 3:**
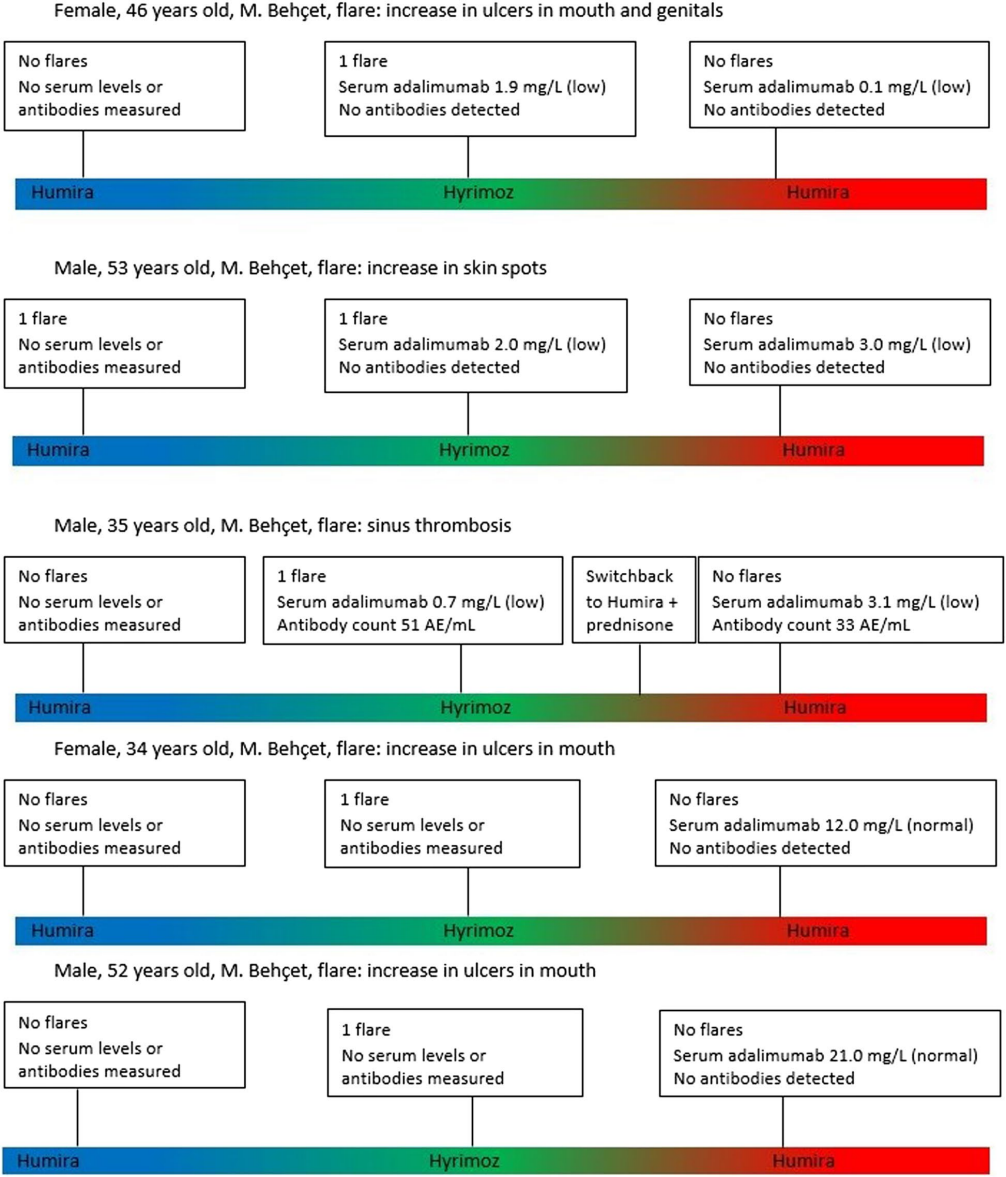
Follow-up of the five patients in which serum adalimumab level and antibodies against adalimumab were measured during Hyrimoz and after switching back to Humira. Blue shows the first period of Humira use, green shows the use of Hyrimoz and red displays the period after switching back to Humira

Among the 5/19 patients who switched from Hyrimoz back to Humira, adalimumab serum levels, and antibodies against adalimumab were measured during follow-up. One patient also received prednisone. Of the five patients, one had anti-drug antibodies, which were lower than those detected during the use of Hyrimoz. In the other four patients, no antibodies were detected before or after switching, considering that antibodies were not measured in two patients before switching. Adalimumab levels measured in five patients after switching back to Humira were within the normal range in 2/5, but these levels were not measured before the switch. In the remaining three patients, the levels were low and remained low after switching after a median follow-up time of 5 (4–9) months (Fig. 3).

## Discussion

This study was conducted to evaluate the real-world effectiveness of the biosimilar adalimumab Hyrimoz, compared to the originator Humira, in the treatment of patients with a variety of IMIDs. We compared the occurrence of flares and adverse effects; there was no significant difference between the occurrence of flares while using Humira and using Hyrimoz, and the number thereof was comparable between Humira and Hyrimoz.

The treatment duration for Humira was longer than that for Hyrimoz because a large proportion of patients stopped Hyrimoz within 1 year of follow-up due to adverse effects or subjective symptoms of the disease (*p < *0.001). A relatively large group of patients treated with biological adalimumab (Humira) and switching to a biosimilar (Hyrimoz) experienced adverse effects or subjective symptoms, leading to early discontinuation of treatment. Therefore, it would be of interest to evaluate the number of patients who discontinue Hyrimoz when starting the biosimilar. Since most biosimilars have been on the market for only a few years, studies on their effectiveness are gradually increasing.

This is the first study to compare the number of flares before and after switching from Humira to Hyrimoz. However, other studies on this topic, with slightly different study designs and patient populations, have shown comparable results. A previous nationwide study in Sweden compared the retention times of biosimilars and their originators, including Humira and Hyrimoz, in patients with rheumatoid arthritis, ankylosing spondylitis, and psoriatic arthritis [24]. The retention times for a biosimilar and its originator were compared after 1 year, showing no differences. In that study, discontinuation was a combined endpoint defined as a lack of effectiveness or an adverse event. Another study evaluating Hyrimoz showed similar effectiveness to another adalimumab biosimilar, Imraldi, in patients with rheumatic diseases [25].

Similar effectiveness was expected based on protein structure. Strikingly, a subgroup of patients in this study developed a flare that was treated effectively by solely changing Hyrimoz back to Humira, without any co-medication. This can be attributed to several factors. It could be that the drug levels of Hyrimoz were low and increased after the change to Humira, either because of low drug levels due to noncompliance or the formation of antibodies. Drug and antibody levels were measured in a minority of patients, showing low levels in three patients at the time of a flare, one of whom had detectable antibody levels. After switching to Humira, drug levels were low during follow-up, and antibodies declined, but were still present. First, the number of follow-up measurements was too small to draw definitive conclusions. Another possibility is the difference in glycosylation between the originator and its biosimilars, resulting in functional changes and changes in the degradation rate.

While no objectified differences in the number of flares were detected between Hyrimoz and Humira, a subgroup of patients in this study switched back to Humira or another biological drug because of a subjective increase in disease activity. This increased activity meant that patients reported more complaints of the disease without being diagnosed with a flare. In most cases, switching back to Humira improved the subjective health experiences of these patients. The increase of subjective symptoms after switching from the originator to the biosimilar has been described before [26]. The possibility of a biosimilar nocebo effect could be considered, even though this is difficult to prove [27]. Discontinuation of Hyrimoz because of subjective symptoms is in line with a previous study, showing subjective symptoms were one of the reasons for discontinuation of a biosimilar [28].

In addition to subjective disease activity, adverse effects were a major reason for discontinuing Hyrimoz therapy. In particular, twenty-five (13.5%) patients reported pain caused by the Hyrimoz injection. Patients specified that this pain was not present while using Humira. In some cases, this pain was very severe, leading to a switch back to Humira. Therefore, the pain associated with Hyrimoz injection is a major concern, as it was the most reported adverse effect. A possible explanation is that citrate, which is known to cause pain upon injection, was used in its preparation. Another contributing factor could be the larger volume of Hyrimoz (50 mg/ml, 0,8 ml per injection) compared to Humira (100 mg/ml; 0,4 ml per injection), which must be injected subcutaneously and may be more painful [29].

Moreover, in a sub-analysis comparing the group in which the disease flared while using Hyrimoz and the group that did not develop a flare while using Hyrimoz, the painfulness of the Hyrimoz injection was reported to be significantly higher. It might be speculated that these patients did not inject Hyrimoz properly or at the right frequency because of the pain associated with these injections.

A quarter of the study population suffered from adverse effects during the use of Hyrimoz. These adverse effects occurred specifically with Hyrimoz and were not present with Humira. As the group that experienced adverse effects comprised almost a quarter of the total cohort, adverse effects were, in general, a major issue with the use of Hyrimoz. However, adverse effects including skin and injection site reactions have been reported with the use of Humira [30]. Humira injections contain citrate and an additive. The painfulness of the injection decreased when citrate was no longer used as an additive. Nash et al. showed that adalimumab without citrate resulted in better tolerability to injections and fewer skin-related side effects [31]. If citrate could be replaced in Hyrimoz, it might be a solution to prevent painful injections.

This study has some limitations. First, the sample size was small. This is because this study was conducted in only one hospital, with a patient population from only one outpatient clinic. A larger cohort may have provided more accurate insights into the effectiveness of Hyrimoz.

This study included of a large group of different disease entities. The primary outcome parameter, a flare, was defined according to the underlying disease. In patients with uveitis, international standards were used to define a flare. For other diseases a flare was objectived as much as possible according to the discretion of the immunologist. Ideally all disease entities would have been reported separately however because of the rarity of most diseases this was not feasible.

Furthermore, because Hyrimoz is a new drug that has not been used in clinical practice for a long time, the immunologists that treated this cohort may have been careful with Hyrimoz. In the case of subjective symptoms reported by patients, they may have switched back to Humira too quickly. In addition, because of painful adverse effects, a relatively large group of patients switched back to Humira, leading to a shorter follow-up period during Hyrimoz. Although the number of flares before and after switching was comparable, the shorter treatment duration of Hyrimoz hampered definite conclusions on effectiveness. However, it is clear that adverse effects, including painful injections, were the main reasons for the switch back to Humira.

Adverse effects events of adalimumab ( Humira) could not be evaluated prospectively. Therefore we chose to describe the adverse effects in the biosimilar group in a descriptive way.

In the questionnaire it was verified whether any reported side effects were already present before switch to the biosimilar or any side effects were reported to the treating physician before switch.

In a subgroup of patients with a flare during Hyrimoz use, inactive disease was achieved by switching to Humira without additional immunosuppressant medication. This could not be explained completely because serum drug and antibody levels before and after switching were only measured in a minority of patients. This is a limitation of this retrospective analysis. Therefore, future research should include both serum drug and antibody measurements to ensure more objective results.

## Conclusion and recommendations

In conclusion, the number of flares during Hyrimoz is comparable to that of its predecessor Humira, and it is, therefore, as effective as Humira from that perspective. However, while using Hyrimoz, a number of patients reported an increase in subjective symptoms, and a quarter of the patients reported side effects. Subjective symptoms of disease activity were the reason for switching back to Humira in a subgroup of patients, after which the symptoms disappeared. Moreover, a quarter of the study population reported adverse effects that specifically developed after switching to Hyrimoz or were related to the use of Hyrimoz (e.g., painful injection). Recommendations for the future are that serum levels and antibodies should be closely monitored in the case of a flare and thereafter.

## Supplementary Information

Supplementary Material 1.

## Data Availability

Data are available on request.

## Abbreviations

IMIDs: Immune-Mediated Diseases
DMARDs: Disease-Modifying Anti-rheumatic Drugs
SUN: Standardization of Uveitis Nomenclature
