# Supplementary material for: Impact of switching from the originator adalimumab to a biosimilar: a retrospective cohort study
Source: BMC Immunol. 2025 Jul 3;26:44. doi: 10.1186/s12865-025-00693-9 (PMC12224804; doi:10.1186/s12865-025-00693-9)
Supplement: Supplementary file 1 — Supplementary Material 1. [file 12865_2025_693_MOESM1_ESM.pdf]

Welkom bij deze vragenlijst. Sinds dit jaar wordt in het Erasmus MC het merk Hyrimoz voorgeschreven (als vervanging van Humira). Hierover zouden we graag uw ervaring horen met deze vragenlijst. Het beantwoorden van deze vragenlijst zal gemiddeld 5-10 minuten in beslag nemen.

Sectie A: Datum en duur gebruik

A1. **In welke maand in 2022 is Hyrimoz voor het eerst bij u toegediend?**

A2. **Weet u de exacte datum van eerste toediening nog? Zo ja, vul de datum in.**

A3. **Gebruikt u Hyrimoz nog steeds?**

Ja ☐

Nee ☐

A4. **Indien nee, in welke maand bent u gestopt?**

A5. **Weet u de exacte datum van uw laatste toeding nog, voordat u bent gestopt? Zo ja, vul de datum in.**

A6. **Waarom bent u gestopt?**

Opvlamming van de ziekte ☐

Bijwerking(en) ☐

Andere reden, namelijk: ☐

Andere reden, namelijk:

Sectie B: Klachten

B1. **Bemerkte u verandering van de ziekte activiteit, nadat u overgestapt was naar Hyrimoz?**

Ja ☐

Nee ☐

B2. **Zijn de klachten van de ziekte erger of minder erg geworden?**

De klachten zijn erger geworden ☐

De klachten zijn minder erg geworden ☐

Sectie C: Bijwerkingen

C1. **Heeft u bijwerkingen van Hyrimoz?**

Ja ☐

Nee ☐

C2. **Heeft u bijwerkingen van Hyrimoz gehad?**

Ja ☐

Nee ☐

C3. **Welke bijwerking is dit? U mag meerdere bijwerkingen noemen, maar u hoeft niet 8 klachten in te vullen.**

Wat is de klacht?:

Klacht 1 ☐☐☐☐☐☐☐☐☐☐

Klacht 2 ☐☐☐☐☐☐☐☐☐☐

Klacht 3 ☐☐☐☐☐☐☐☐☐☐

Klacht 4 ☐☐☐☐☐☐☐☐☐☐

Klacht 5 ☐☐☐☐☐☐☐☐☐☐

Klacht 6 ☐☐☐☐☐☐☐☐☐☐

Klacht 7 ☐☐☐☐☐☐☐☐☐☐

Klacht 8 ☐☐☐☐☐☐☐☐☐☐

Had u deze klacht al voordat u Hyrimoz bent gaan gebruiken?:

Klacht 1 ☐☐☐☐☐☐☐☐☐☐

Klacht 2 ☐☐☐☐☐☐☐☐☐☐

Klacht 3 ☐☐☐☐☐☐☐☐☐☐

Klacht 4 ☐☐☐☐☐☐☐☐☐☐

Klacht 5 ☐☐☐☐☐☐☐☐☐☐

Klacht 6 ☐☐☐☐☐☐☐☐☐☐

Klacht 7 ☐☐☐☐☐☐☐☐☐☐

Klacht 8 | | | | | | | | | |

Zo ja, is de klacht toegenomen, afgenomen of gelijk gebleven sinds dat u Hyrimoz bent gaan gebruiken?:

Klacht 1

Klacht 2

Klacht 3

Klacht 4

Klacht 5

Klacht 6

Klacht 7

Klacht 8

Sectie D: Ervaring met Hyrimoz

D1. Wat vond u van de verandering naar Hyrimoz?

D2. Zijn er nog andere dingen die u wilt zeggen over uw verandering naar Hyrimoz?

Sectie E: Algemene vragen

E1. Wat is uw leeftijd?

E2. Wat is uw geslacht?

- Man ☐
- Vrouw ☐
- Non-binair ☐
- Zeg ik liever niet ☐

E3. Wat is uw hoogst afgeronde opleiding?

- Basisonderwijs ☐
- Vmbo kader ☐
- Vmbo basis ☐
- Vmbo-t ☐
- Havo ☐
- Vwo ☐
- Mbo ☐
- Hbo ☐
- WO bachelor ☐
- WO master ☐
- Doctoraat ☐
- Anders, namelijk: ☐

Anders, namelijk:

E4. In welk land bent u geboren?

- Nederland ☐
- Anders, namelijk: ☐

Anders, namelijk:

E5. Zijn uw ouders in Nederland geboren?

- Ja ☐
- Nee ☐

E6. Zo nee, waar zijn uw ouders dan geboren?

Bedankt voor het beantwoorden van de vragenlijst. U kunt mogelijk nog gebeld worden, als wij aanvullende vragen hebben. Als u hier liever niet aan mee doet, dan kunt u dat als u gebeld wordt direct aangeven. U hoeft geen reden op te geven.
